# Significant subgraph mining for neural network inference with multiple comparisons correction

**DOI:** 10.1162/netn_a_00288

**Published:** 2023-06-30

**Authors:** Aaron J. Gutknecht, Michael Wibral

**Affiliations:** Department of Data-driven Analysis of Biological Networks, Göttingen Campus Institute for Dynamics of Biological Networks, Georg August Universtiy, Göttingen, Germany; Johann-Friedrich-Blumenbach Institute, Georg August University, Göttingen, Germany; Brain Imaging Center, Goethe University, Frankfurt am Main, Germany

**Keywords:** Graph theory, Statistics, Multiple comparisons, Network inference, Transfer entropy, Autism

## Abstract

We describe how the recently introduced method of significant subgraph mining can be employed as a useful tool in neural network comparison. It is applicable whenever the goal is to compare two sets of unweighted graphs and to determine differences in the processes that generate them. We provide an extension of the method to dependent graph generating processes as they occur, for example, in within-subject experimental designs. Furthermore, we present an extensive investigation of the error-statistical properties of the method in simulation using Erdős-Rényi models and in empirical data in order to derive practical recommendations for the application of subgraph mining in neuroscience. In particular, we perform an empirical power analysis for transfer entropy networks inferred from resting-state MEG data comparing autism spectrum patients with neurotypical controls. Finally, we provide a Python implementation as part of the openly available IDTxl toolbox.

## Author Summary

A key objective of neuroscientifc research is to determine how different parts of the brain are connected. The end result of such investigations is always a graph consisting of nodes corresponding to brain regions or nerve cells and edges between the nodes indicating if they are connected or not. The connections may be structural (an actual anatomical connection) but can also be functional, meaning that there is a statistical dependency between the activity in one part of the brain and the activity in another. A prime example of the latter type of connection would be the information flow between brain areas. Differences in the patterns of connectivity are likely to be responsible for and indicative of various neurological disorders such as autism spectrum disorders. It is therefore important that efficient methods to detect such differences are available. The key problem in developing methods for comparing patterns of connectivity is that there is generally a vast number of different patterns (it can easily exceed the number of stars in the milky way). In this paper we describe how the recently developed method of significant subgraph mining accounts for this problem and how it can be usefully employed in neuroscientific research.

## INTRODUCTION

Comparing networks observed under two or more different conditions is a pervasive problem in network science in general, and especially in neuroscience. A fundamental question in these cases is if the observed patterns or motifs in two samples of networks differ solely due to chance or because of a genuine difference between the conditions under investigation. For example, a researcher may ask if a certain pattern of functional connections in a brain network reconstructed from magnetoencephalography (MEG) data is more likely to occur in individuals with autism spectrum disorder than in neurotypic controls, or whether an observed difference in occurrence is solely due to chance. What makes this question difficult to answer is the fact that the number of possible patterns in the network scales as 2^*l*^2^^, with *l* the number of network nodes, leading to a formidable multiple comparison problem. Correcting for multiple comparisons with standard methods (e.g., Bonferroni) typically leads to an enormous loss of power, as these methods do not exploit the particular properties of the network comparison problem.

By contrast, the recently developed significant subgraph mining approach (Llinares-López, Sugiyama, Papaxanthos, & Borgwardt, 2015; Sugiyama, López, Kasenburg, & Borgwardt, 2015) efficiently solves the network-comparison problem while maintaining strict bounds on type I error rates for between unit of observation designs. Within the landscape of graph theoretic methods in neuroscience the distinguishing features of subgraph mining are, first, that it works with binary graphs; second, that it does not rely on summary statistics such as average clustering, modularity, or degree distribution (for review see, for instance, Bullmore & Sporns, 2009); and third, that it is concerned with the *statistical* differences between graph generating processes rather than the distance between two individual graphs (for examples of such graph metrics see Mheich et al., 2017; Schieber et al., 2017; Shimada, Hirata, Ikeguchi, & Aihara, 2016). Subgraph mining can be considered the most fine grained method possible for the comparison of binary networks in that it is in principle able to detect *any* statistical difference.

Here we describe how to adapt this method to the purposes of network neuroscience and provide a detailed study of it’s error-statistical properties (family-wise error rate and statistical power) in both simulation and empirical data. In particular, we describe an extension of subgraph mining for within unit of observation designs that was, to our best knowledge, not described in the literature before. Furthermore, we utilize Erdős-Rényi networks as well as an empirical dataset of transfer entropy networks to investigate the behaviour of the method under different network sizes, sample sizes, and connectivity patterns. Based on these analyses, we discuss practical recommendations for the application of subgraph mining in neuroscience. Finally, we provide an openly available implementation of subgraph mining as part of the python toolbox IDTxl (https://github.com/pwollstadt/IDTxl; Wollstadt et al., 2019). The implementation readily deals with various different data structures encountered in neuroscientific research. These include directed and undirected graphs, between- and within-subject designs, as well as data with or without a temporal structure.

In the following section, we will explain the core ideas behind the original subgraph mining method as introduced in Llinares-López et al. (2015) and Sugiyama et al. (2015), putting an emphasis on concepts and intuition, but also providing a rigorous mathematical exposition for reference. We then turn to the extension for within-subject designs before presenting the simulation-based and empirical investigation of subgraph mining.

## BACKGROUND AND THEORY: THE ORIGINAL SUBGRAPH MINING METHOD

**Figure 1.**
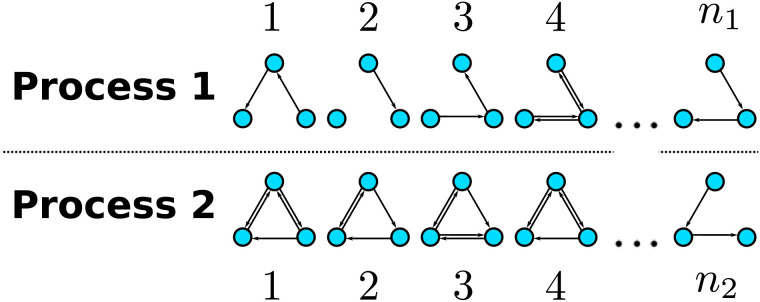
Illustration of two graph-generating processes. Each process consists of randomly sampling individuals from a specific population and describing the neural activity of these individuals as a graph. The population underlying process 1 is sampled *n*_1_ times and the population underlying process 2 is sampled *n*_2_ times. The nodes may correspond to different brain areas while the edges describe any directed relationship between brain areas such as information transfer.

Neural networks can usefully be described as graphs consisting of a set of nodes and a set of edges connecting the nodes (Bullmore & Sporns, 2009). The nodes represent specific parts of the network such as individual neurons, clusters of neurons, or larger brain regions, whereas the edges represent relationships between these parts. Depending on whether the relationship of interest is symmetric (such as correlation) or asymmetric (such as transfer entropy or Granger causality), the network can be modelled as an undirected or as a directed graph, respectively. Once we have a decided upon an appropriate graph theoretic description, we can apply it to networks measured in two different experimental groups, resulting in two sets of graphs. In doing so, we are essentially sampling from two independent graph-generating processes (see Figure 1 for illustration).

**Figure 2.**
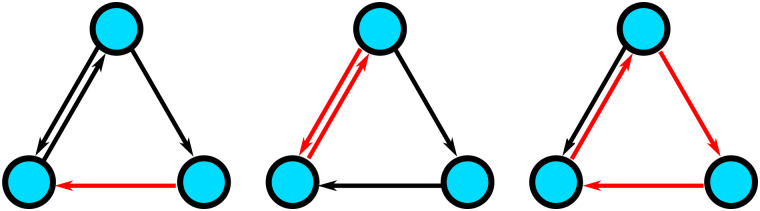
Illustration of subgraphs with one edge (left), two edges (middle), and three edges (right) of a graph with three nodes.

The key question is now if there are any significant differences between these two sets. However, since graphs are complex objects it is not immediately obvious how they should be compared. In principle, one may imagine numerous different possibilities. For instance, comparing the average number of connections of a node or the average number of steps it takes to get from one node to another. Instead of relying on such summary statistics, however, one may also take a more fine-grained approach by looking for differences with respect to any possible pattern, or more technically subgraph, that may have been observed. Does a particular edge occur significantly more often in one group than in the other? What about particular bidirectional connections? Or are there even more complex subgraphs—consisting of many links—that are more frequently observed in one of the groups? Answering such questions affords a particularly detailed description of the differences between the two processes. Figure 2 shows examples of different subgraphs of a graph with three edges.

The process of enumerating all subgraphs for which there is a significant difference between the groups is called significant subgraph mining (Sugiyama et al., 2015). The goal is to identify all subgraphs that are generated with different probabilities by the two processes. The main difficulty underlying significant subgraph mining is that the number of possible subgraphs grows extremely quickly with the number of nodes. For a directed graph with seven nodes, it is already in the order of 10^14^. This not only imposes runtime constraints but also leads to a severe multiple comparisons problem. Performing a significance test for each potential subgraph and then adjusting by the number of tests is not a viable option because the resulting test will have an extremely poor statistical power. As will be detailed later, due to the discreteness of the problem the power may even be exactly zero because *p* values low enough to reach significance can in principle not be achieved. In the following sections we will describe the original (between-subjects) significant subgraph mining method developed by Llinares-López et al. (2015) and Sugiyama et al. (2015) by first setting up an appropriate probabilistic model, explaining how to construct a significance test for a particular subgraph, and finally, detailing two methods for solving the multiple comparisons problem.

### Probabilistic Model

We are considering two independently sampled sets of directed graphs 𝒢_1_ and 𝒢_2_ describing, for instance, connections between brain regions in two experimental groups. Each set contains one graph per subject in the corresponding group and we assume that the (fixed) sample sizes of each group are *n*_1_ = |𝒢_1_| and *n*_2_ = |𝒢_2_|. All graphs are defined on the same set of nodes *V* = {1, 2, …, *l*} but may include different sets of links (edges) *E* ⊆ *V* × *V*. The graphs are assumed to have been generated by two potentially different graph-generating processes. Each process can be described by a random *l* × *l* adjacency matrix of, possibly dependent, Bernoulli random variables:

$$X^{\left(k\right)}=\left[\begin{array}{cccc} X_{11}^{\left(k\right)} & X_{12}^{\left(k\right)} & \ldots & X_{1l}^{\left(k\right)}\\ X_{21}^{\left(k\right)} & X_{22}^{\left(k\right)} & \ldots & X_{1l}^{\left(k\right)}\\ \ldots & \ldots & \ldots & \ldots \\ X_{1l}^{\left(k\right)} & \ldots & \ldots & X_{ll}^{\left(k\right)} \end{array}\right]$$ (1)

where the superscript *k* = 1, 2 indicates the group and

$$X_{ij}^{\left(k\right)}∼\text{Bern}\left(p_{ij}^{\left(k\right)}\right),\;1\leq i,j\leq l$$ (2)

Each of those variables tells us whether the corresponding link from node *i* to node *j* is present (“1”) or absent (“0”). A graph-generating process can be fully characterized by the probabilities with which it generates possible subgraphs. Specifically, there is one such probability for each possible graph *G* = (*V*, *E*_*G*_) on the nodes under consideration. The probability that *G* occurs as a subgraph of the generated graph in group *k* is given by

$$\pi_{G}^{\left(k\right)}=\mathbb{P}\left(\underset{\left(i,j\right)\in E_{G}}{\cap }\left\{X_{ij}^{\left(k\right)}=1\right\}\right)$$ (3)

where (*i*, *j*) indicates an individual link from node *i* to node *j*. It is important to note that $\pi_{G}^{\left(k\right)}$ denotes the probability that all the edges of *G* are realized plus possibly some additional edges. This is to be distinguished from the probability that *exactly* the graph *G* is realized. In the following we will always refer to the probability $\pi_{G}^{\left(k\right)}$ as the subgraph probability of *G*. A graph generating process is completely specified when all it’s subgraph probabilities are specified. So to sum up, we can model the two sets of directed graphs 𝒢_1_ and 𝒢_2_ as realizations of two independent graph generating processes **X**^(1)^ and **X**^(2)^. Process **X**^(1)^ generates graphs according to subgraph probabilities $\pi_{G}^{\left(1\right)}$ whereas the subgraph probabilities for process **X**^(2)^ are given by $\pi_{G}^{\left(2\right)}$. Based on this probabilistic model we may now proceed to test for differences between the two processes.

### Testing Individual Subgraphs

Our goal now is to find those subgraphs *G* that are generated with different probabilities by the two processes. If the two processes describe two distinct experimental groups, this means that we are trying to identify subgraphs whose occurrence depends on group membership. Thus, for each possible subgraph *G*, we are testing the null hypothesis of *equal subgraph probabilities*, or equivalently, of *independence of subgraph occurrence from group membership*

$$H_{0}^{G}:\pi_{G}^{\left(1\right)}=\pi_{G}^{\left(2\right)}$$ (4)

against the alternative of unequal subgraph probabilities or dependence on group membership

$$H_{1}^{G}:\pi_{G}^{\left(1\right)}\neq \pi_{G}^{\left(2\right)}$$ (5)

In order to test such a null hypothesis, we have to compare how often the subgraph *G* occurred in each group and determine if the observed difference could have occurred by chance, that is, if the probability of such a difference would be larger than the significance level *α* under the null hypothesis. The relevant data for this test can be summarized in a 2 × 2 contingency table (Table 1). Where *f*_*i*_(*G*) denotes the *observed* absolute frequency of subgraph *G* in Group *i*, *f*(*G*) = *f*_1_(*G*) + *f*_2_(*G*) denotes the *observed* absolute frequency of *G* in the entire data set, and *n* = *n*_1_ + *n*_2_ is the total sample size.

**Table 1.** Contingency table

**Subgraph G**	Occurrences	Nonoccurrences	Total
Group 1	*f*_1_(*G*)	*n*_1_ − *f*_1_(*G*)	*n*_1_
Group 2	*f*_2_(*G*)	*n*_2_ − *f*_2_(*G*)	*n*_2_
Total	*f*(*G*)	*n* − *f*(*G*)	*n*

In the following, we will use *F*_*i*_(*G*) and *F*(*G*) to denote the corresponding *random* absolute frequencies. Given our model assumptions above, the numbers of occurrences in each group are independent Binomial variables: on each of the *n*_1_ (or *n*_2_) independent trials there is a fixed probability $\pi_{G}^{\left(1\right)}$ (or $\pi_{G}^{\left(2\right)}$) that the subgraph *G* occurs. This means that our goal is to compare two independent Binomial proportions. This can be achieved by utilizing Fisher’s exact test (Llinares-López et al., 2015; Sugiyama et al., 2015) which has the advantage that it does not require any minimum number of observations per cell in the contingency table.

**Figure 3.**
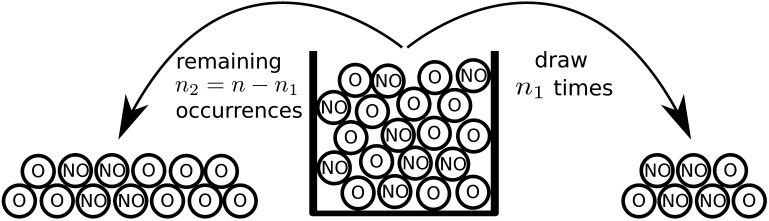
Comparing two Binomial proportions using Fisher’s exact test. Under the null hypothesis and conditional on the total number of occurrences of a subgraph, the occurrences are distributed over the groups as if drawn at random without replacement out of an urn containing one ball per subject. The balls are labelled ‘O’ if the subgraph occurred in the corresponding subject and ‘NO’ if it did not occur. In the illustration *n* = 20 (number of total measurements, balls), *n*_1_ = 7 (number of measurements for group 1), and *f*(*G*) = 12 (number of occurences, balls with ‘O’). The seven balls drawn for group 1 are shown to the right of the urn. They include three occurrences and four nonoccurrences. This result would lead to an insignificant *p* value of ≈0.5.

The key idea underlying Fisher’s exact test is to condition on the total number of occurrences *f*(*G*). Specifically, the random variable *F*_1_(*G*) can be shown to follow a hypergeometric distribution under the null hypothesis and conditional on the total number of occurrences. In other words, if the null hypothesis is true and given the total number of occurrences, the *n*_1_ occurrences and nonoccurrences of subgraph *G* in Group 1 are assigned as if they were drawn randomly without replacement out of an urn containing exactly *f*(*G*) occurrences and *n* − *f*(*G*) nonoccurrences (see Figure 3). *F*_1_(*G*) can now be used as a test statistic for the hypothesis test.

Since we are interested in differences between the graph-generating processes in either direction, the appropriate test is a *two-sided* one. For a right-sided test of the null hypothesis against the alternative $\pi_{G}^{\left(1\right)}$ > $\pi_{G}^{\left(2\right)}$ the *p* value can be computed as

$$p_{G}^{R}=\sum_{k=f_{1}\left(G\right)}^{\min\left(f\left(G\right),n_{1}\right)}hyp\left(k;N,f\left(G\right),n_{1}\right)$$ (6)

summing up the probabilities of all possible values of *f*_1_(*G*) *larger than or equal to* the one actually observed. Note that *f*_1_(*G*) cannot be larger than *min*(*f*(*G*), *n*_1_) because the number of occurrences in Group 1 can neither be larger than the sample size *n*_1_ nor larger than the total number of occurrences *f*(*G*). A left-sided *p* value can be constructed analogously. The two-sided test rejects the null hypothesis just in case the two-sided *p* value

$$p_{G}=2*\text{min}\left(p_{G}^{L},p_{G}^{R}\right)$$ (7)

is smaller than or equal to *α*.

### Multiple Comparisons

Since there may be a very large number of possible subgraphs to be tested we are faced with a difficult multiple comparisons problem. For a directed graph with seven nodes the number of possible subgraphs is already in the order of 10^14^. If we were to use this number as a Bonferroni correction factor, the testing procedure would have an exceedingly low statistical power, meaning that it would be almost impossible to detect existing differences in subgraph probabilities. In the following, we will describe two methods for solving the multiple comparisons problem: the Tarone correction (Tarone, 1990) and the Westfall-Young permutation procedure (Westfall & Young, 1993), which have been used in the original exposition of significant subgraph mining by Llinares-López et al. (2015) and Sugiyama et al. (2015).

#### Tarone’s correction.

**Figure 4.**
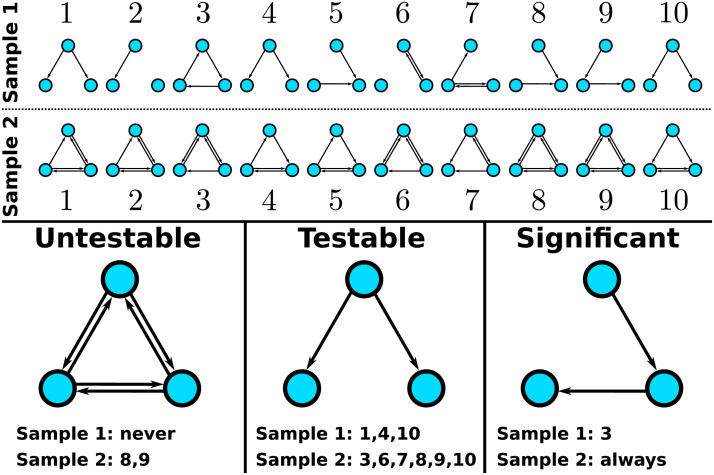
Examples of 0.05 untestable, 0.05 testable, and significant subgraphs for a data set consisting of 10 graphs per group (top panel). The fully connected graph is untestable at level 0.05 because it only occurs twice in the data set (group 2 samples 8 and 9) leading to a minimum achievable *p* value of ≈0.47. The graph shown on the bottom middle is testable at level 0.05 since it occurs nine times in total. This means that its minimum achievable *p* value is ≈0.0001. However, it is not significant with an actual (uncorrected) *p* value of ≈0.37. The graph shown on the bottom right reaches significance using Tarone’s correction factor *K*(0.05) = 17. It occurs every time in group 2 but only once it group 1, which results in a corrected *p* value of ≈0.02.

The subgraph mining problem is discrete in the sense that there is only a finite number of possible *p* values. This fact can be exploited to drastically reduce the correction factor. The key insight underlying the Tarone correction is that given any total frequency *f*(*G*) of a particular subgraph *G* there is a *minimum achievable p value* which we will denote by $p_{G}^{*}$. Intuitively, this minimum achievable *p* value is reached if the *f*(*G*) occurrences are distributed as unevenly as possible over the two groups. We may now introduce the notion of the set *T*(*k*) of $\frac{\alpha }{k}$-testable subgraphs:

$$T\left(k\right)=\left\{G\subseteq G_{C}:p_{G}^{*}\leq \frac{\alpha }{k}\right\}$$ (8)

containing all subgraphs whose minimum achievable *p* value is smaller than or equal to $\frac{\alpha }{k}$. Following Tarone, the number of elements of this set can be denoted by *m*(*k*) = |*T*(*k*)|. Tarone et al. then showed that the smallest integer *k* such that $\frac{m\left(k\right)}{k}$ ≤ 1 is a valid correction factor in the sense that the probability of rejecting a true null hypothesis, the family-wise error rate (FWER), is bounded by *α* (Tarone, 1990). Moreover, the family-wise error rate is controlled no matter which or how many null hypotheses are true (see Supporting Information for proof). This property is called strong control. A slight improvement of this correction factor was proposed by Hommel and Krummenauer (1998) (for details see Supporting Information). Figure 4 illustrates the concepts of testable, untestable, and significant subgraphs.

#### Westfall-Young correction.

The familiy-wise error rate with respect to a corrected significance level *δ* can be expressed in terms of the cumulative distribution function of the smallest *p* value associated with a true null hypothesis: the event that there is at least one false positive is identical with the event that the smallest *p* value associated with a true null hypothesis is smaller than *δ*. The same applies to the *conditional* family-wise error rate given the total occurrences of each graph in the dataset:

$$\text{CFWER}\left(\delta \right)=\mathbb{P}\left(\min_{G\in {𝒢}_{0}}\left(P_{G}\right)\leq \delta |F=f\right)$$ (9)

where 𝒢_0_ is the set of subgraphs for which the null is true and *F* is the vector of the total occurrences of each subgraph. This means that if the correction factor is chosen as the *α*-quantile of the distribution in Equation 9 the family-wise error rate is controlled. The problem is that we cannot evaluate the required distribution because we don’t know which hypotheses are true. The idea underlying the Westfall-Young correction is to instead define the correction factor as the *α*-quantile of the distribution of the minimal *p* value across *all* subgraphs and under the *complete* null-hypothesis (stating that all null hypotheses are true). This correction factor always provides weak control of the FWER in the sense that the FWER is bounded by *α* under the complete null-hypothesis (the issue of strong control is addressed in the Discussion section). It can be estimated via permutation strategies. The procedure is as follows: First, we may represent the entire dataset by Table 2.

**Table 2.** Representation of the entire data set

Subject	Group	*G*_1_	*G*_2_	…	*G*_*m*_
1	0	0	1	…	1
2	0	1	1	…	1
…	…	…	…	…	…
*n*_1_	0	0	0	…	1
*n*_1_ + 1	1	1	1	…	1
…	…	…	…	…	…
*n*_1_ + *n*_2_	1	0	1	…	1

The columns labelled *G*_*i*_ tell us if subgraph *G*_*i*_ was present or absent in the different subjects (rows). The column labelled ”Group” describes which group the different subjects belong to. Under the complete null hypothesis the group labels are arbitrarily exchangeable. This is because, given our independence assumptions, all the observed graphs in the dataset are independent and identically distributed samples from the same underlying distribution in the complete null case. The column of group labels is now shuffled, reassigning the graphs in the dataset to the two groups. Based on this permuted dataset we can recompute a *p* value for each *G*_*i*_ and determine the smallest of those *p* values. Repeating this process many times allows us to obtain a good estimate of the distribution of the smallest *p* value under the complete null hypothesis. The Westfall-Young correction factor is then chosen as the *α*-quantile of this permutation distribution. Since the number of permutations grows very quickly with the total sample size, it is usually not possible to evaluate all permutations. Instead, one has to consider a much smaller random sample of permutations in order to obtain an approximation to the permutation distribution. This procedure can be shown to be valid as long as the identity permutation (i.e., the original dataset) is always included (Hemerik & Goeman, 2018).

**Figure 5.**
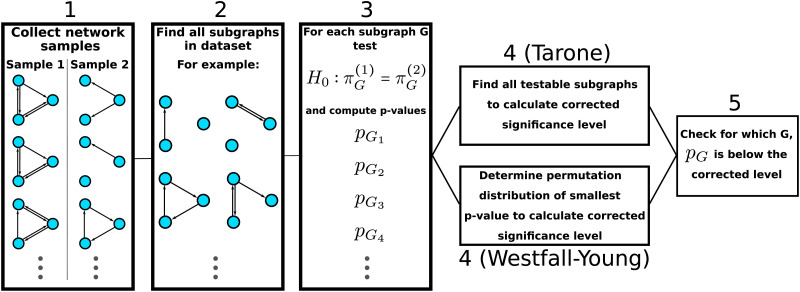
Schematic illustration of significant subgraph mining. Note that for computational efficiency various shortcuts can be employed. The figure describes conceptually how significant subgraph mining works rather than it’s fastest possible implementation (see, e.g., Llinares-López et al. (2015), for a fast algorithm implementing the Westfall-Young correction).

This concludes our discussion of the original subgraph mining method. Figure 5 provides a schematic illustration of the essential steps. In the next section, we describe how the method can be extended to be applicable to within-subject experimental designs, which abound in neuroscience.

## EXTENSION TO WITHIN-SUBJECT DESIGNS

So far we have considered networks associated with subjects from two groups and we assumed that the numbers of occurrences of a subgraph in the two groups are independent of each other. However, there are many cases in which there is only a single group of subjects and we are interested in how the networks differ between two experimental conditions. Since the same subjects are measured in both conditions, the independence assumption is not warranted anymore. Because Fisher’s exact test assumes independence, the approach described above has to be modified. In particular, in case of dependence, the null distribution of the number of occurrences in the first group/condition will in general not be a hypergeometric distribution potentially leading to inflated type I error rates in Fisher’s exact test. An appropriate alternative is McNemars test for marginal homogeneity. It essentially tests the same null hypothesis as Fisher’s exact test, but is based on a wider probabilistic model of the graph generating processes. In particular, the independence assumption is relaxed, allowing for dependencies between the two experimental conditions: whether a subgraph occurs in condition A in a particular subject may affect the probability of its occurrence in condition B and vice versa. Suppose we are observing *n* subjects in two conditions. We may denote the random adjacency matrices corresponding the *i*-th subject in condition 1 and 2 by $X_{i}^{\left(1\right)}$ and $X_{i}^{\left(2\right)}$, respectively. Then the probabilistic model for the graph-generating processes is:

$$\left(\begin{array}{c} X_{1}^{\left(1\right)}\\ X_{1}^{\left(2\right)} \end{array}\right),\left(\begin{array}{c} X_{2}^{\left(1\right)}\\ X_{2}^{\left(2\right)} \end{array}\right),\ldots ,\left(\begin{array}{c} X_{n}^{\left(1\right)}\\ X_{n}^{\left(2\right)} \end{array}\right)\;i.i.d.$$ (10)

For each subject there is an independent and identically distributed realization of the two graph-generating processes. The two processes themselves may be dependent since they describe the same subject being observed under two conditions. The distributions of $X_{i}^{\left(1\right)}$ and $X_{i}^{\left(2\right)}$ are again determined by the subgraph probabilities $\pi_{G}^{\left(1\right)}$ and $\pi_{G}^{\left(2\right)}$ and for any particular *G* we would like to test the null hypothesis:

$$H_{0}^{G}:\pi_{G}^{\left(1\right)}=\pi_{G}^{\left(2\right)}$$ (11)

The idea underlying McNemar’s test is to divide the possible outcomes for each subject into four different categories: (1) *G* occurred in both conditions, (2) *G* occurred in neither condition, (3) *G* occurred in condition 1 but not in condition 2, (4) *G* occurred in condition 2 but not in condition 1. The first two categories are called *concordant pairs* and the latter two are called *discordant pairs*. The discordant pairs are of particular interest because differences in subgraph probabilities between the two conditions will manifest themselves in the relative number of the two types of discordant pairs: If $\pi_{G}^{\left(1\right)}$ > $\pi_{G}^{\left(2\right)}$, then we would expect to observe the outcome ‘*G* occurred only in condition 1’ more frequently than the outcome ‘*G* occurred only in condition 2’. Conversely, if $\pi_{G}^{\left(2\right)}$ > $\pi_{G}^{\left(1\right)}$, than we would expect to observe the latter type of discordant pair more frequently. The frequency of any of the four categories can be represented in a contingency table (Table 3).

**Table 3.** Contingency table

**Condition 1 / Condition 2**	Yes	No	Total
Yes	$Y_{11}^{G}$	$Y_{10}^{G}$	*F*_1_(*G*)
No	$Y_{01}^{G}$	$Y_{00}^{G}$	*n* − *F*_1_(*G*)
Total	*F*_2_(G)	*n* − *F*_2_(*G*)	*n*

The variables $Y_{11}^{G}$, $Y_{11}^{G}$, $Y_{21}^{G}$, $Y_{22}^{G}$ are the counts of the four categories. The numbers of occurrences in each condition *F*_1_(*G*) and *F*_2_(*G*) appear in the margins of the contingency table. McNemar’s test uses the upper right entry, $Y_{10}^{G}$, as the test statistic. Conditional on the total number of discordant pairs, $Y_{10}^{G}$ + $Y_{01}^{G}$, and under the null hypothesis, this test statistic has a binomial distribution

$$Y_{10}^{G}|Y_{10}^{G}+Y_{01}^{G}=d\overset{H_{0}}{∼}Bin\left(d,\frac{1}{2}\right)$$ (12)

If there are exactly *d* discordant pairs and the probability of *G* is equal in both conditions, then both types of discordant pairs (‘only in condition 1’ or ‘only in condition 2’) occur independently with equal probabilities in each of the d subjects where a discordant pair was observed. A two-sided test can be constructed in just the same way as described above for the between-subjects case. First, we construct right- and left-sided *p* values as:

$$p_{G}^{L}=\sum_{k=0}^{y_{10}^{G}}Bin\left(k;d,\frac{1}{2}\right)\;p_{G}^{R}=\sum_{k=y_{10}^{G}}^{d}Bin\left(k;d,\frac{1}{2}\right)$$ (13)

Then the two-sided *p* value is

$$p_{G}=2*\min\left(p_{G}^{L},p_{G}^{R}\right)$$ (14)

Exactly like the Fisher’s test, McNemar’s test also has a minimal achievable *p* value. The only difference is that it is not a function of the total number of occurrences in condition A, but a function of the number of discordant pairs. The Tarone correction described above remains valid if Fisher’s exact test is simply replaced by McNemar’s test. The Westfall-Young procedure requires some modifications because the permutation strategy described above is not valid anymore. The problem is that, because of possible dependencies between the conditions, condition labels are not arbitrarily exchangeable under the complete null hypothesis. Instead we have to take a more restricted approach and only exchange condition labels *within subjects*. In doing so, we are not only keeping the total number of occurrences *F*(*G*) constant for each subgraph, but also the total number of discordant pairs *D*(*G*). Accordingly, the theoretical Westfall-Young correction factor, estimated by the modified permutation strategy, is the *α*-quantile of the conditional distribution of the smallest *p* value given *F* = *f* and *D* = *d* and under the complete null hypothesis (where *F* and *D* are the vectors of total occurrences and discordant pair counts for all subgraphs).

## VALIDATION OF MULTIPLE COMPARISONS CORRECTION METHODS USING ERDŐS-RÉNYI MODELS

In this section we empirically investigate the family-wise error rate and statistical power of the multiple comparison correction methods for significant subgraph mining described above. In doing so we will utilize Erdős-Rényi models for generating random graphs. In these models the edges occurs independently with some common probability *p*_*i*_ in each graph-generating process. This means that the subgraph probability for a graph *G* = (*V*, *E*_*G*_) in process *i* is *p*_*i*_ raised to the number of edges *G* consists of:

$$\pi_{G}^{\left(i\right)}=p_{i}^{\left|E_{G}\right|}$$ (15)

If *p*_*i*_ is the same for both graph-generating processes (*p*_1_ = *p*_2_), then the complete null hypothesis is satisfied. By contrast, if *p* is chosen differently for the two processes (*p*_1_ ≠ *p*_2_), then the null hypothesis of equal subgraph probabilities is violated for all subgraphs, that is, the *complete alternative* is satisfied. We used the former setting for the purposes of FWER estimation and the latter for power analysis. Furthermore, the two graph-generating processes were simulated independently of each other which corresponds to the between-subjects case. Accordingly, Fisher’s exact test was used throughout.

### Family-Wise Error Rate

**Figure 6.**
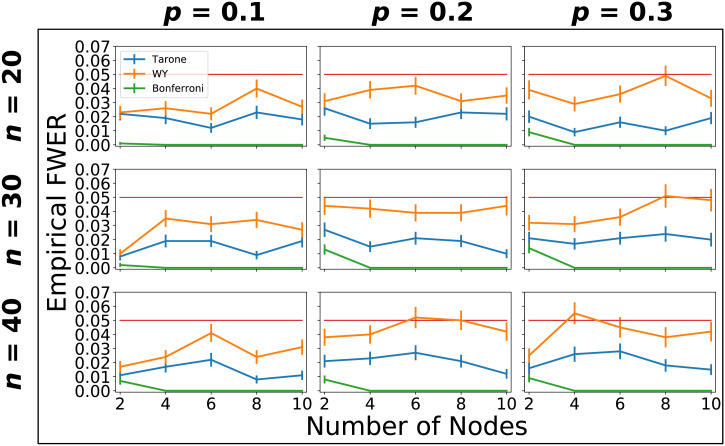
Estimated family-wise error rates of Tarone, Westfall-Young, and Bonferroni corrections based on 1,000 simulations and different sample sizes, connection densities, and network sizes. Error bars represent one standard error. The estimated FWER never exceeded the desired FWER of *α* = 0.05 (red horizontal line) by more than one standard error for all correction methods. In fact, it was always smaller than 0.05 except in three cases for the Westfall-Young correction (0.051, 0.052, and 0.055). The estimated FWERs of the three methods were always ordered in the same way: the Bonferroni correction had the smallest estimated FWER (at most 0.014), followed by the Tarone correction (at most 0.028), and the Westfall-Young correction (at most 0.055).

In order to empirically ascertain that the desired bound on the family-wise error rate is maintained by the Tarone and Westfall-Young corrections in the subgraph mining context, we performed a simulation study based on Erdős-Rényi models. We tested sample sizes *n* = 20, 30, 40, network sizes *l* = 2, 4, 6, 8, 10, and connection densities *p* = 0.1, 0.2, 0.3. For each combination of these values we carried out 1,000 simulations and estimated the empirical FWER as the proportion of simulations in which one or more significant subgraphs were identified. Figure 6 shows the results of this analysis. The FWER is below the prespecified *α* = 0.05 in all cases for the Tarone and Bonferroni corrections and always within one standard error of this value for the Westfall-Young correction. The Bonferroni correction is most conservative. In fact, the FWER quickly drops to exactly zero since the Bonferroni-corrected level is smaller than the smallest possible *p* values. The Tarone correction reaches intermediate values of 0.1–0.3 while the Westfall-Young correction is always closest the prespecified level and sometimes even reaches it.

### Power

We now turn our attention to the statistical power of the multiple comparison correction methods, that is, their ability to detect existing differences between subgraph probabilities. Previous studies have used the empirical FWER as a proxy for statistical power (Llinares-López et al., 2015; Sugiyama et al., 2015). The rationale underlying this approach is that the more conservative a method is (i.e. the more the actual FWER falls below the desired significance level), the lower its statistical power. In the following we will take a more direct approach and evaluate the performance of the methods under the alternative hypothesis of unequal subgraph probabilities. Again we will utilize Erdős-Rényi models, only now with different connection densities *p*_1_ ≠ *p*_2_ for the two graph-generating processes. The question is: How many subgraphs are we able to correctly identify as being generated with distinct probabilities by the two processes? The answer to this question will not only depend on the multiple comparisons correction used but also on the sample size, the network size, and the effect size. The effect size *for a particular subgraph G* can be identified with the magnitude of the difference of subgraph probabilities |$\pi_{G}^{\left(1\right)}$ − $\pi_{G}^{\left(2\right)}$|. The larger this difference, the better the chances to detect the effect. In the following, we will use the difference between the connection densities *p*_1_ and *p*_2_ as a measure of the effect size for the entire graph-generating processes.

**Figure 7.**
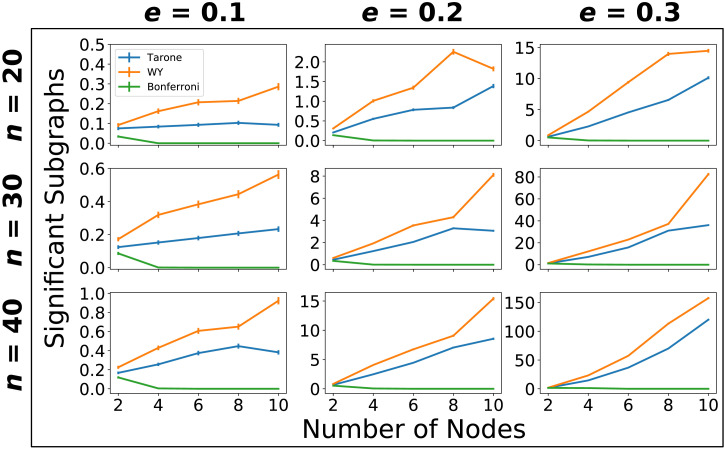
Average number of significant subgraphs identified depending on correction method, samples size, network size, and effect size. Error bars represent one standard error. The number of identified subgraphs increases with sample size (rows) and effect size (columns) for all correction methods.

In a simulation study we analyzed sample sizes *n* = 20, 30, 40. We set the probability of individual links for the first graph-generating process to *p*_1_ = 0.2. The second process generated individual links with probability *p*_2_ = 0.2 + *e*, where *e* = 0.1, 0.2, 0.3. Since *p*_1_ and *p*_2_ are chosen smaller than or equal to 0.5, the effect sizes for particular subgraphs are a decreasing function of the number of edges they consist of. In other words, the difference is more pronounced for subgraphs consisting only of few edges and can become very small for complex subgraphs. We considered network sizes *l* = 2, 4, 6, 8, 10. For each possible choice of *n*, *e*, and *l* we simulated 1,000 datasets and applied significant subgraph mining with either Tarone, Westfall-Young, or Bonferroni correction. The number of permutations for the Westfall-Young procedure was set to 10,000 as recommended in previous studies (Llinares-López et al., 2015). The two graph-generating processes were sampled independently (between subjects case) and accordingly Fisher’s exact test was utilized. The results are shown in Figure 7.

As expected the average number of detected significant subgraphs is an increasing function of both sample size and effect size. The relationship between detected differences and number of nodes is less straightforward. Generally, there is an increasing relationship, but there are a few exceptions. The likely explanation for this phenomenon is that there is a trade-off between two effects: on the one hand, the larger the number of nodes the more differences there are to be detected. But on the other hand, the larger the number of nodes the more severe the multiple comparisons problem becomes which will negatively affect statistical power. For some parameter settings this latter effect appears to be dominant. The most powerful method is always the Westfall-Young correction followed by the Tarone correction. The Bonferroni correction has the worst performance and its power quickly drops to zero because the corrected threshold can in principle not be attained.

Generally, only a very small fraction of existing differences is detectable. Since the graphs are generated by independently selecting possible links with a fixed probability, the subgraph probability is a decreasing function of the number of links a subgraph consists of. Complex subgraphs are quite unlikely to occur and will therefore not be testable. Additionally, the difference between subgraph probabilities $\pi_{G}^{\left(1\right)}$ and $\pi_{G}^{\left(2\right)}$ decreases with increasing subgraph complexity making this difference more difficult to detect. For instance, if *e* = 0.3, then the difference in subgraph probabilities for subgraphs with 10 nodes is about 0.001. Accordingly, even with a sample size of 40, only a small fraction of existing differences is detectable.

## EMPIRICAL POWER ANALYSIS WITH TRANSFER ENTROPY NETWORKS

**Figure 8.**
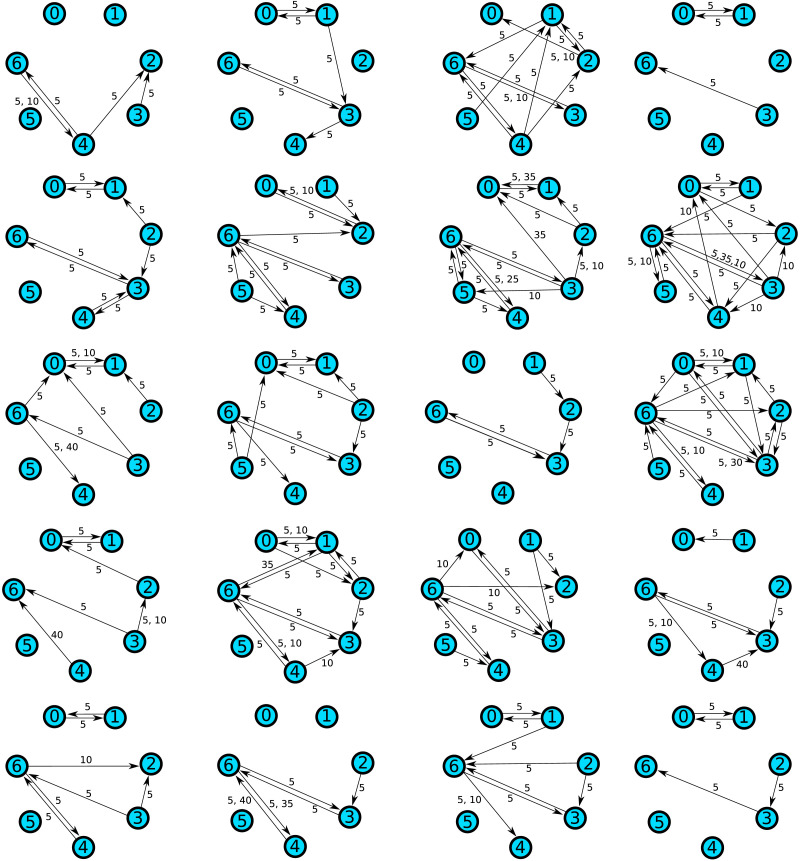
Transfer entropy networks detected in autism spectrum group.

**Figure 9.**
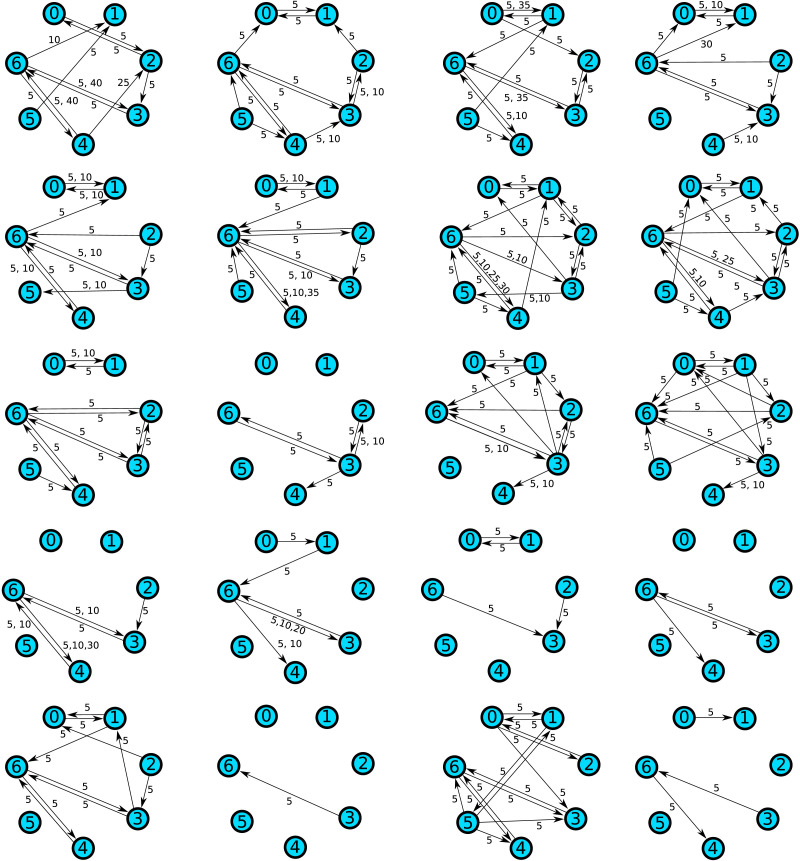
Transfer entropy networks detected in control group.

We applied the subgraph mining method to a dataset of resting-state MEG recordings comparing 20 autism spectrum disorder patients to 20 neurotypical controls. The details of the study are described in Brodski-Guerniero et al. (2018). Here, seven voxels of interest were identified based on differences in local active information storage; subsequently time courses of neural mass activity in these voxels were reconstructed by means of a linear constraint minimum variance beamformer. The locations of the voxels are shown in Table 4. We applied an iterative greedy method to identify multivariate transfer entropy networks on these voxels (Lizier & Rubinov, 2012; Novelli, Wollstadt, Mediano, Wibral, & Lizier, 2019). This is at present considered the best (Novelli & Lizier, 2021) means of measuring neural communication in data (also called “communication dynamics”) (Avena-Koenigsberger, Misic, & Sporns, 2018). The goal of this method is to find for each target voxel a set of source voxels such that (1) the total transfer entropy from the sources to the target is maximized, and (2) each source provides significant transfer entropy conditional on all other source voxels in the set. The outcome of this procedure is one directed graph per subject where each link represents significant information transfer from one voxel to another (conditional on the other sources). Accordingly, we are in a setting in which subgraph mining is applicable. The inferred transfer entropy graphs are shown in Figure 8 and Figure 9. Note that the edges are labeled by numbers that represent the time lags at which information transfer occurred. The parameters of the network inference algorithm were chosen so that lags are always multiples of five. Since the sampling rate was 1200 Hz, this corresponds to a lag increment of ≈4 ms. So the graph representation also contains information about the temporal structure of information transfer and differences in this structure can be detected by subgraph mining as well. For example, even if the probability of detecting information transfer from voxel 0 to voxel 1 is the same in both groups, this transfer may be more likely to occur at a time lag of 5 (≈4 ms) in the autism group, whereas it may be more likely to occur at a time lag of 10 (≈8 ms) in the control group.

**Table 4.** Voxel IDs and corresponding brain regions

Voxel ID	Location
0	Cerebellum
1	Cerebellum
2	Lingual Gyrus / Cerebellum
3	Posterior Cingulate Cortex (PCC)
4	Precuneus
5	Supramarginal Gyrus
6	Precuneus

We applied subgraph mining with both Tarone and Westfall-Young correction to this dataset. No significant differences between the ASD group and control group could be identified. Due to the rather small sample size, this result is not entirely unexpected. For this reason, we performed an empirical power analysis in order to obtain an estimate of how many subjects per group are required in order to detect existing differences between the groups. This estimate may serve as a useful guideline for future studies. The power analysis was performed in two ways: first, by resampling links independently using their empirical marginal frequencies, and second, by resampling from the empirical joint distribution, that is, randomly drawing networks from the original data sets with replacement.

**Figure 10.**
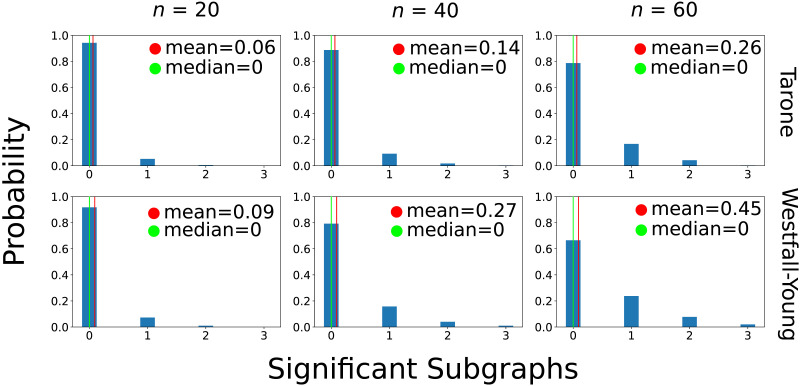
Results of empirical power analysis assuming *independence* of links. We simulated sample sizes 20, 40, and 60 per group and carried out 400 simulations in each setting. The histograms describe the fractions of simulations in which different numbers of significant subgraphs were detected.

The results of the power analysis assuming independent links are shown in Figure 10. We simulated sample sizes 20, 40, and 60 per group and carried out 400 simulations for each setting. The first notable outcome is that the original data are strikingly different from the results seen in independent sampling of links. In particular, the number of testable graphs is far higher in the original data (1,272) than in the independently resampled data (28.7 on average and 55 at most). This indicates strongly that the processes generating the networks in ASD patients as well as controls do not generate links independently. Rather, there seem to be dependencies between the links such that some links tend to occur together, making it more likely that subgraphs consisting of these links will reach testability. Accordingly, in the case of independent resampling, much larger sample sizes are needed in order to detect the differences between the groups. Even in the *n* = 60 per group setting there were only 0.26 (Tarone) and 0.45 (Westfall-Young) significant subgraphs on average. There was no simulation in which more than three significant subgraphs were detected.

**Figure 11.**
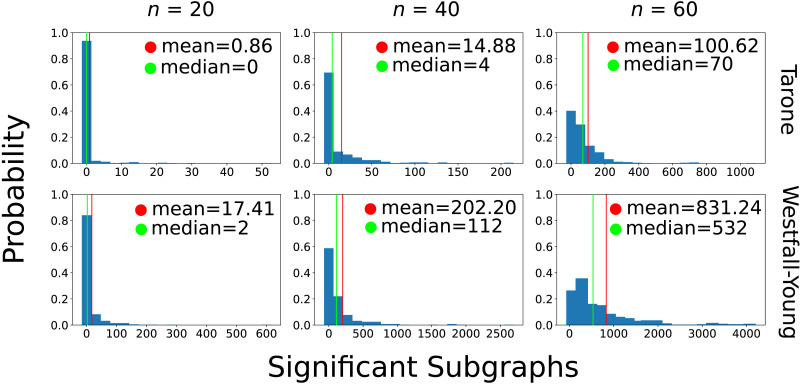
Results of empirical power analysis performed by sampling from the empirical joint distribution. We simulated sample sizes 20, 40, and 60 per group and carried out 400 simulations in each setting. The histograms describe the fractions of simulations in which different numbers of significant subgraphs were detected.

The simulation results of the empirical power analysis based on the empirical joint distribution are shown in Figure 11. Again we used sample sizes 20, 40, and 60. The average number of testable subgraphs is in the same order of magnitude as in the original data set for the *n* = 20 setting (≈5,200). Moreover, the number of identified significant subgraphs is far greater than in independent sampling for all sample sizes. The Westfall-Young correction identifies more subgraphs on average than the Tarone correction: 17.41 compared to 0.86 for *n* = 20, 202.20 compared to 14.88 for *n* = 40, and 831, 24 compared to 100.62 for *n* = 60. The distributions are always highly skewed with more probability mass on smaller values. This is reflected in the median values also shown in the figure. For example, notwithstanding the average value of 14.88 significant subgraphs in the *n* = 40 setting with Tarone correction, the empirical probability of not fining any significant subgraph is still ≈42%. For the Westfall-Young correction this probability is only ≈1.8% in the *n* = 40 setting. In the *n* = 60 setting both methods have high empirical probability to detect significant differences. In fact, the Westfall-Young correction always found at least one difference, and the Tarone correction only failed to find differences in 2.5% of simulations. The total number of detected differences can be in the thousands in this setting.

Since in the *n* = 60 setting both methods are likely to detect some of the existing differences, we performed a subsequent analysis to narrow down the effect sizes that can be detected in this case. For each possible effect size (any multiple of 0.05 up to 0.35), we enumerated all subgraphs with this effect size and calculated their empirical detection probabilities among the 400 simulations. In total, there were about 3.7 million subgraphs occurring with different empirical probabilities in the two groups. Most of these (99.5%) are subgraphs that occur exactly once in the entire dataset. One important reason for this phenomenon is the following: suppose a network contains a subgraph that occurs only once in the dataset. Then removing any other edges or combination of edges from the network will again result in a subgraph that only occurs once in the data set. Consider for example the last network in the second row in Figure 8. It contains a connection from node 6 to node 3 at a lag of 35 samples. This connection does not occur in any other network. This means that if any combination of the other 18 links occurring in the network is removed, the result will again be a uniquely occurring subgraph. There are 2^18^ = 262,144 possibilities for doing so in this case alone.

**Figure 12.**
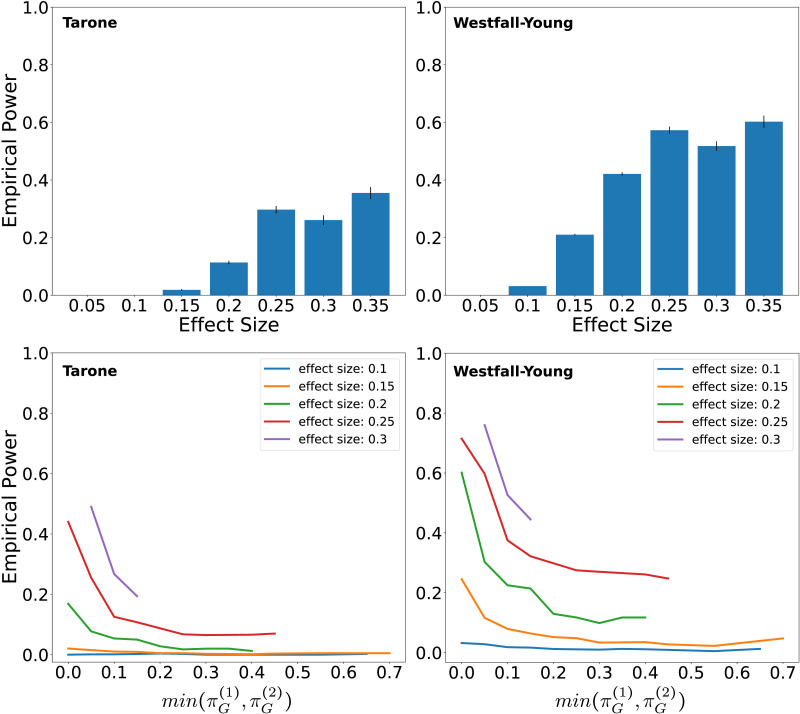
Empirical power versus effect size (*n* = 60). Upper plots: Average empirical detection probabilities for subgraphs with different effect sizes (i.e., the average is over all subgraphs with a certain effect size and for each particular graph the detection probability is estimated as the fraction of detection among the 400 simulations). Error bars are plus minus one standard error. Standard errors were not calculated for effect size 0.05 due to computational constraints. There are more than 3.7 million subgraphs with this effect size meaning that in the order of 10^12^ detection covariances would have to be computed. This is necessary because the detections of different subgraphs are not independent. However, due to this large number of subgraphs, the standard errors are bound to be exceedingly small in this case. Lower plots: Dependence of average detection probability on minimum of the two subgraph occurrence probabilities for different effect sizes. Even subgraphs with the same effect size have considerably different detection probabilities depending on how extreme the absolute occurrence probabilities are.

The averages of the empirical detection probabilities for each effect size are shown in Figure 12 (upper plots). An interesting outcome is that the detection probability is not a strictly increasing function of the effect size. Rather there is a slight drop from effect sizes 0.25 to 0.3. Given the standard errors of the estimates, this result might still be explained by statistical fluctuation (the two standard error intervals slightly overlap). However, in general this type of effect could also be real because the effect size is not the only factor determining detection probability. This is illustrated in Figure 12 (lower plots), which shows average detection probability over the smaller of the two occurrence probabilities *min*($\pi_{G}^{\left(1\right)}$, $\pi_{G}^{\left(2\right)}$). It turns out that the more extreme this probability is, the more likely the effect is to be detected. The highest detection probability is found if the empirical probability of occurrence is zero in one of the groups. For this reason it can in fact be true that the detection probability is on average higher for effect sizes of size 0.25 than 0.3, if the absolute occurrence probabilities are more extreme in the former case. In the data analyzed here this is in fact the case: roughly half of the subgraphs with effect size 0.25 do have occurrence probability zero in one of the groups, whereas this is not true for any of the subgraphs with effect size 0.3.

## DISCUSSION

### What Are the Appropriate Application Cases for Subgraph Mining?

A key feature of significant subgraph mining that distinguishes it from other statistical methods for graph comparisons is that it considers all possible differences between graph-generating processes. In other words, as soon as these processes differ in any way, subgraph mining is guaranteed to detect these differences if the sample size is large enough. This is in contrast to methods that only consider particular *summary statistics* of the graph generating processes such as the average degree of a node. Such methods are of course warranted if there is already a hypothesis about a specific summary statistic. For example, Viol et al. (2019) were specifically interested in the entropy of the distribution of shortest paths from a given node to a randomly chosen second node. In such a case, performing a statistical test with respect to the statistic of interest is preferable over subgraph mining because the multiple comparisons problem is avoided. This leads to a higher statistical power *regarding the statistic in question*. On the other hand, the test will have a low power to detect other differences between the processes. There are also well-known methods such as the network-based statistic (NBS) developed by Zalesky, Fornito, and Bullmore (2010) operating on a more fine-grained level than summary statistic approaches. NBS aims to identify significant differences with respect to certain “connected components” of links. Thus, in terms of localizing resolution it is in between a summary statistic analysis and a full link-by-link comparison. Again, there is a trade-off here between statistical power with respect to certain features of the graph generating processes on the one hand and resolution on the other. Compared to a method specifically tailored toward a particular summary statistic, the NBS will likely be less powerful. But due to its higher localizing resolution it will be able to detect differences toward which the summary statistic is blind.

Subgraph mining is on the far end of localizing resolution when it comes to comparing binary graph-generating processes (by contrast NBS works with weighted graphs). Even if the two processes generate any individual link with the same probability, there may be differences in terms of dependencies or interactions between link occurrences. These will be reflected in different subgraph probabilities for more complex subgraphs, and subgraph mining is guaranteed to detect these differences given a sufficiently large sample. Of course, this comes at the price of having to deal with a very severe multiple comparisons problem. However, it would not be correct to say that for this reason subgraph mining has lower statistical power than more coarse-grained alternatives. Rather one should say that increasing the localizing resolution will always come at the price of a lower statistical power *with respect to certain differences*, but at the same time it will increase statistical power with respect to those differences that are only visible at the higher resolution. Given it’s extremely high resolution, we propose that subgraph mining should be the method of choice if no hypothesis about some specific difference between the graph-generating processes is available so that no custom-tailored tests of those differences can be applied. In such a case, subgraph mining can be utilized to systematically explore the entire search space of all possible differences.

### What Are the Requirements on Sample Size?

The appropriate sample size depends primarily on the kinds of effect sizes one seeks to be able to detect. Our empirical power analysis of the MEG dataset discussed in the previous section suggests that in similar studies a sample size of about 60 is sufficient to have a very high probability to detect at least some of the existing differences. We carried out an additional analysis in order to narrow down the effect sizes likely to be detected at this sample size. This analysis showed that the largest effect sizes occurring in the empirical joint distribution (≈0.35 difference in probability of occurrence) had a detection probability of ≈0.4 on average using the Tarone correction and ≈0.6 on average using the Westfall-Young correction. This means that for a *particular* graph with a certain effect size the probability of detecting it is not extremely high. However, since there is generally a large number of such graphs there is a high probability of detecting at least some of them. Our analysis also showed that the effect size, understood as the difference in probability of occurrence of a subgraph between the groups, is not the only factor determining statistical power. Even graphs with the same effect size can have different probabilities of detection depending on how extreme the absolute probabilities of occurrence are. The detection probability is particularly high if the occurrence probability of a subgraph is close to zero in one of the groups. By symmetry we also expect this to be the case if it is close to one.

A possible way to reduce the amount of data required is to restrict the subgraph mining to subgraphs *up to a prespecified complexity*. For example, one could perform subgraph mining for all possible subgraphs consisting of up to three links. The validity of the method is not affected by this restriction. However, the search space is reduced and hence the multiple comparisons problem becomes less severe. In applying subgraph mining in this way it is important to prespecify the desired complexity. Otherwise, we would run into yet another multiple comparisons problem. Consider the MEG dataset presented in the previous section. Upon not detecting any differences with the full subgraph mining algorithm, which considers all subgraphs on the seven nodes in our networks, one could check for differences among subgraphs consisting of at most six nodes. If nothing is found here either, we could move on to five nodes and so forth until we are down to a single link comparison. However, this approach would not be valid because the individual links are essentially given seven chances to become significant, so that our bounds on the family-wise error rate do not hold anymore.

### What Are the Computational Costs of Subgraph Mining?

Besides the required sample size another factor for the applicability of subgraph mining is the computation time. The number of possible subgraphs can very easily be large enough that it becomes impossible to carry out a test for each one of them. Of course, the main idea behind the multiple comparisons methods presented here is that a large number of subgraphs can be ignored because they do not occur often enough or too often to be testable. For how many subgraphs this is true depends in particular on the connection density of the graphs. Generally, the computational load be will greater the more nodes the graphs consist of and the more densely these nodes are connected. However, if the graphs are extremely densely connected one could revert to the *negative* versions of the graphs, which would in this case be very loosely connected.

We provide a python implementation of significant subgraph mining as part of the IDTxl toolbox (https://github.com/pwollstadt/IDTxl; Wollstadt et al., 2019). It offers both Tarone (with or without Hommel improvement) and Westfall-Young corrections. The latter is implemented utilizing the “Westfall-Young light” algorithm developed by Llinares-López et al. (2015), which we also adapted for within-subject designs. Details on the computational complexity can be found in this reference as well. The algorithm performs computations across permutations and achieves substantially better runtimes than a naive permutation-by-permutation approach. Our implementation is usable for both between-subjects and within-subject designs and allows the user to specify the desired complexity of graphs up to which subgraph mining is to be performed (see previous paragraph). It is also able to take into account the temporal network structure as described in the application to transfer entropy networks.

### Which Multiple Comparisons Correction Method Should Be Used?

The choice between the two multiple comparison correction methods is a matter of statistical power on the one hand and a matter of false-positive control guarantees on the other. Regarding power, the Westfall-Young correction clearly outperforms the Tarone correction. Regarding false-positive control the situation is more complicated: whereas the Tarone correction is proven to control the family-wise error rate in the strong sense, the Westfall-Young procedure *in general* only provides weak control (see Westfall & Young, 1993). There is, however, a sufficent (but not necessary) condition for strong control of the Westfall-Young procedure called *subset pivotality*. Formally, a vector of *p* values **P** = (*P*_1_, *P*_2_, …, *P*_*m*_) has subset pivotality if and only if for any subset of indices *K* ⊆ {1, 2, …, *m*} the joint distribution of the subvector *P*_*K*_ = {*P*_*i*_|*i* ∈ *K*} is the same under the restrictions ∩_*i*∈*K*_ *H*_0*i*_ and ∩_*i*∈{1,…,*m*}_ *H*_0*i*_ (Dudoit, van der Laan, & Pollard, 2004; Westfall & Young, 1993). In the subgraph mining context this means that the joint distribution of *p* values corresponding to subgraphs for which the null hypothesis is in fact true remains unchanged in the (possibly counterfactual) scenario that the null hypothesis is true for *all* subgraphs. It has been stated in the literature that the subset pivotality condition is not particularly restrictive and holds under quite minimal assumptions (Westfall & Troendle, 2008). However, to the best of our knowledge, it has not yet been formally established in the subgraph mining context. A future study addressing this issue would therefore be highly desirable.

Just to clarify the practical role of the distinction between weak and strong control, weak-control does not allow a *localization* of differences between graph generating processes. It only warrants the conclusion that there must be *some* difference. The reason is essentially the same as the reason why it is not warranted to reject a null hypotheses if it’s *p* value has not been corrected for multiple comparisons at all. Suppose we perform 20 tests at level 0.05 and a particular null hypothesis, say the fifth one, turns out to reach significance. If we did not correct for multiple comparisons, it would be a mistake to reject the fifth hypothesis because there is a plausible alternative explanation for why it reached significance: because we did not control for having performed 20 tests, it was to be expected that we would see at least one hypothesis rejected and it just *happened* to be the fifth one. Similarly, if we only have weak control of the FWER and a particular subgraph, say *G*_5_, reaches significance, then it would be a mistake to conclude that *G*_5_ is actually generated with different probabilities by the two processes. The alternative explanation is that our false positive probabilities are not controlled under the actual scenario (the ground truth) and *G*_5_ simply happened to turn out significant. The only scenario that weak control *does* rule out (and this is how it differs from not controlling at all) is the one where all null hypotheses are true, that is, the one where the two graph generating processes are identical.

## CONCLUSION

Significant subgraph mining is a useful method for neural network comparison especially if the goal is to explore the entire range of possible differences between graph-generating processes. The theoretical capability to detect any existing stochastic difference is what distinguishes subgraph mining from other network comparison tools. Based on our empirical power analysis of transfer entropy networks reconstructed from an MEG dataset we suggest to use a sample size of at least 60 subjects per group in similar studies. The demand on sample size and computational resources can be reduced by carrying out subgraph mining only up to a prespecified subgraph complexity or by reverting to the negative versions of the networks under consideration. The method can also be used for dependent graph-generating processes arising in within-subject designs when the individual hypothesis tests and multiple comparisons correction methods are appropriately adapted. We provide a full Python implementation as part of the IDTxl toolbox that includes these functionalities.

## ACKNOWLEDGMENTS

We thank Lionel Barnett for helpful discussions on the topic.

## SUPPORTING INFORMATION

Supporting information for this article is available at https://doi.org/10.1162/netn_a_00288.

## AUTHOR CONTRIBUTIONS

Aaron Julian Gutknecht: Conceptualization; Formal analysis; Investigation; Methodology; Software; Validation; Visualization; Writing – original draft; Writing – review & editing. Michael Wibral: Conceptualization; Data curation; Project administration; Resources; Supervision; Writing – review & editing.

## FUNDING INFORMATION

Michael Wibral, Volkswagen Stiftung, Award ID: Big Data in den Lebenswissenschaften. Michael Wibral, Niedersächsisches Ministerium für Wissenschaft und Kultur (https://dx.doi.org/10.13039/501100010570), Award ID: Niedersächsisches Vorab. Michael Wibral, Volkswagen Stiftung, Award ID: Niedersächsisches Vorab. Michael Wibral, Deutsche Forschungsgemeinschaft, Award ID: CRC 1193 C04. We acknowledge support by the Open Access Publication Funds of Göttingen University.

## TECHNICAL TERMS

Graph-generating process: A random process that generates binary graphs according to certain probabilities.
Subgraph: Any motif or pattern of connections on the nodes under consideration. Generically denoted by *G*. In practice, only those patterns that actually occur in the dataset are relevant. But in order to fully characterize the underlying graph-generating processes, all possible patterns have to be considered.
Significant subgraph mining: The procedure of determining all subgraphs whose number of occurrence is statistically significantly different in two groups/conditions.
Subgraph probability: The probability with which a subgraph/pattern is generated as part of a network (to be distinguished from the probability that exactly the pattern in question in generated).
Testable subgraph: A subgraph is testable at a given significance level just in case the most extreme allocation of its occurrences over the groups/conditions leads to a significant *p* value.
Family-wise error rate (FWER): The probability of erroneously rejecting at least one null hypothesis.
Strong control: A multiple comparisons correction provides strong control if it bounds the family-wise error rate by the predefined significance level *α* no matter which or how many null hypotheses are in fact true.
Weak control: A multiple comparisons correction provides weak control if it bounds the family-wise error rate by the predefined significance level *α* under the assumption that all null hypotheses are true.
Transfer entropy: A measure of the information flow between two stochastic processes. It quantifies the degree to which the prediction of a target process is improved by taking into account the history of a source process relative to predicting the target only based on its own history.
