# Supplementary material for: Significant subgraph mining for neural network inference with multiple comparisons correction
Source: Netw Neurosci. 2023 Jun 30;7(2):389–410. doi: 10.1162/netn_a_00288 (PMC10312259; doi:10.1162/netn_a_00288)
Supplement: Supplementary file 1 [file netn-7-2-389-s001.pdf]

Gutknecht, A. J. & Wibral, M. (2022). Supporting information for "Significant subgraph mining for neural network inference with multiple comparisons correction." *Network Neuroscience.* Advance publication. https://doi.org/10.1162/netn_a_00288.

Journal: **NETWORK NEUROSCIENCE**

METHODS

Supporting Information for Significant Subgraph Mining for Neural Network Inference with Multiple Comparisons Correction

Aaron J. Gutknecht[1,2,3], **Michael Wibral**[1,2]

[1]Dept. Data-driven Analysis of Biological Networks, Göttingen Campus Institute for Dynamics of Biological Networks, Georg August Universtiy, Göttingen, Germany

[2]Johann-Friedrich-Blumenbach Institute, Georg August University, Göttingen, Germany

[3]Brain Imaging Center, Goethe University, Frankfurt am Main, Germany

PROOF OF VALIDITY OF TARONE'S CORRECTION FACTOR

The validity of the Tarone correction factor $K(\alpha)$ can be seen as follows: Let $\mathcal{G}_0$ denote the set of subgraphs for which the null hypothesis of equal subgraph probabilities is true and let $T_0(k) = \{G \in \mathcal{G}_0 | p^*_G \leq \frac{\alpha}{k}\}$ be the subset of $\frac{\alpha}{k}$-testable subgraphs within $\mathcal{G}_0$. Furthermore, let $m_0(k)$ be the number of elements of this set, i.e. the number of $\frac{\alpha}{k}$-testable subgraphs for which the null-hypothesis is true. We can now compute the conditional family-wise error rate for a correction factor $k \in \mathbb{N}$ given the observed total frequencies of each subgraph. These frequencies can be interpreted as the realization of a random vector F containing one entry $F(G)$ per possible subgraph:

$$CFWER\left(\frac{\alpha}{k}\right) = \mathbb{P}\left(\bigcup_{G \in \mathcal{G}_0} \{p_G \leq \frac{\alpha}{k}\} \mid F = f\right) \tag{1}$$

We only have to take the union over $\frac{\alpha}{k}$-testable subgraphs because all other terms have probability zero:

$$= \mathbb{P}\left(\bigcup_{G \in T_0(k)} \{p_G \leq \frac{\alpha}{k}\} \mid F = f\right) \tag{2}$$

Using Boole's inequality (the "union bound"):

$$\leq \sum_{G \in T_0(k)} \mathbb{P}\left(p_G \leq \frac{\alpha}{k} \mid F = f\right) \tag{3}$$

By construction of the p-value we have for any constant $c \in \mathbb{R}^+ : \mathbb{P}\left(p_G \leq c | F = f\right) \leq c$. This fact can be applied to each term in the above sum with $c = \frac{\alpha}{k}$:

$$\leq \sum_{G \in T_0(k)} \frac{\alpha}{k} \tag{4}$$

The sum has $m_0(k)$ terms:

$$= m_0(k) \frac{\alpha}{k} \tag{5}$$

The number of testable subgraphs for which the null-hypothesis is true is smaller than or equal to the total number of testable subgraphs:

$$\leq m(k) \frac{\alpha}{k} \tag{6}$$

$$\overset{!}{\leq} \alpha \tag{7}$$

For the final equation (6) to be valid it must be true that $\frac{m(k)}{k} \leq 1$. In order to maximize the power of the resulting test, the correction factor should be chosen as small as possible. Hence, the appropriate choice is the smallest integer k such that $\frac{m(k)}{k} \leq 1$, i.e. $K(\alpha)$. Since the argument is valid for all possible observed total frequencies, it is also valid for the unconditional FWER which is simply a weighted average of the conditional FWERs:

$$FWER\left(\frac{\alpha}{K(\alpha)}\right) = \mathbb{P}\left(\bigcup_{G \in \mathcal{G}_0} \{p_G \leq \frac{\alpha}{K(\alpha)}\}\right) \tag{8}$$

$$= \sum_f \mathbb{P}(F = f) \mathbb{P}\left(\bigcup_{G \in \mathcal{G}_0} \{p_G \leq \frac{\alpha}{K(\alpha)}\} \mid F = f\right) \tag{9}$$

$$\leq \alpha \sum_f \mathbb{P}(F = f) \tag{10}$$

$$= \alpha \tag{11}$$

It is important to note that this argument does not make any assumptions about *which* or *how many* null-hypotheses are in fact true. The FWER is controlled in all cases. This property is called *strong control* of the FWER.

HOMMEL IMPROVEMENT OF TARONE'S CORRECTION

The Tarone correction has been criticized on the basis that it is not α-consistent Roth (1999). This means that a null-hypothesis might not be rejected at level α even though it would have been rejected at an even

smaller level $\delta < \alpha$. However, there is a simple modification proposed by Hommel and Krummenauer (1998) that makes the Tarone procedure α-consistent and, maybe more importantly, also improves its statistical power. The idea is to make the procedure α-consistent *by definition*, i.e. to reject H_0^G if the standard Tarone procedure would reject or if there exists a level $\gamma < \alpha$ such that the standard Tarone procedure would reject:

$$\text{Reject } H_0^G \text{ if and only if there exists a } \gamma,\ 0 < \gamma \leq \alpha,\ \text{such that } p_G \leq \frac{\gamma}{K(\gamma)} \quad (12)$$

This rule has to be at least as powerful as the standard Tarone procedure because a null-hypothesis is rejected by the standard procedure it is also rejected by the improved version. Additionally, there are cases in which the Hommel impovement rejects but the standard Tarone procedure does not. Hommel presented a simple algorithm to implement this idea which, in the subgraph mining context, can be phrased as follows: First, we order all subgraphs in terms of their minimal achievable p-values such that $p^*_{G_1} \leq p^*_{G_2} \leq ... \leq p^*_{G_m}$, where $m = 2^{l^2}$ is the total number of possible subgraphs. Then we define the rejection rule as:

$$\text{Reject } H_0^G \text{ if and only if either } p_G \leq \frac{\alpha}{K(\alpha)} \text{ or } p_G < p^*_{G_{K(\alpha)}} \quad (13)$$

REFERENCES

Hommel, G., & Krummenauer, F. (1998). Improvements and modifications of tarone's multiple test procedure for discrete data. *Biometrics*, 673–681.

Roth, A. J. (1999). Multiple comparison procedures for discrete test statistics. *Journal of statistical planning and inference*, *82*(1-2), 101–117.
